# Why don't smallholder farmers in Kenya use more biopesticides?

**DOI:** 10.1002/ps.5896

**Published:** 2020-05-30

**Authors:** Kate L Constantine, Monica K Kansiime, Idah Mugambi, Winnie Nunda, Duncan Chacha, Harrison Rware, Fernadis Makale, Joseph Mulema, Julien Lamontagne‐Godwin, Frances Williams, Steve Edgington, Roger Day

**Affiliations:** ^1^ CABI Egham UK; ^2^ CABI Nairobi Kenya

**Keywords:** smallholder farmer, perceptions, biopesticides, willingness to pay, Kenya

## Abstract

**Background:**

Although Kenya has a relatively high number of registered biopesticide products, little is known about biopesticide use by smallholders. This paper documents farmers' current use and perception of chemical pesticides and biopesticides, their willingness to pay for biopesticides, and the key challenges to biopesticide uptake.

**Results:**

A survey found that chemical pesticides are used widely by smallholders despite awareness of the risks to human health and the environment. Almost half of respondents showed awareness of biopesticides, but current use in the survey localities was low (10%). Key reasons for the low use of biopesticides by smallholders in this study are: perceptions of effectiveness, primarily speed of action and spectrum of activity, availability and affordability. Smallholders who used biopesticides cited effectiveness, recommendation by advisory services and perception of safety as key reasons for their choice. Although farmers viewed both pesticides and biopesticides as costly, they invested in the former due to their perceived effectiveness. Average willingness to pay, above current chemical pesticide expenditures per cropping season was 9.6% (US$5.7). Willingness to pay differed significantly between counties, and was higher among farmers with more education or greater awareness of the health risks associated with pesticide use.

**Conclusion:**

This study confirms the low use of biopesticide products in the survey areas, alongside high use of conventional chemical pesticides. In order to promote greater uptake of biopesticides, addressing farmers' awareness and their perceptions of effectiveness is important, as well as increasing the knowledge of those providing advice and ensuring registered products are available locally at competitive prices. © 2020 The Authors. *Pest Management Science* published by John Wiley & Sons Ltd on behalf of Society of Chemical Industry.

## Graphical abstract

Key reasons for low use of biopesticides by smallholders in this study were perceptions of effectiveness, speed of action and spectrum of activity, availability and affordability.

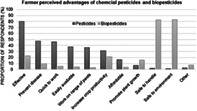

## 1 INTRODUCTION

Agriculture is vital to Kenya's economy, with the sector accounting for 51% of the country's gross domestic product.^1^ The sector is central to food security, poverty reduction and economic growth, with the majority of the poor depending on smallholder agriculture (78% of total production) for their livelihoods.^1^, ^2^

Aside from drought and weather‐related risks, invertebrate pests, diseases and weeds contribute to considerable pre‐ and post‐harvest losses, resulting in reduced yields and incomes, threatening food security and poverty reduction.^2^, ^3^, ^4^ Particularly for higher value crops, many smallholders rely heavily on the use of chemical pesticides to tackle crop pests, despite increased awareness of the potential negative impacts. Pesticides can damage human and animal health,^4^, ^5^, ^6^, ^7^ exacerbated by limited use of personal protective equipment, and have detrimental effects on the environment and biodiversity.^4^, ^5^, ^6^, ^7^, ^8^ Incorrect usage can also lead to the development of pesticide resistance, reducing the cost‐effectiveness of control. Integrated pest management (IPM) has therefore been promoted as the basis for sustainable agriculture, giving priority to ecological, safer methods of crop production, and minimising the use of pesticides.^3^

Biopesticides are potentially important tools in IPM, with fewer drawbacks than chemical pesticides. The term ‘biopesticide’ covers a broad range of active ingredients including macro‐organisms (e.g. predatory and parasitic insects and mites), micro‐organisms (bacteria, fungi, viruses), botanical extracts, semiochemicals and secondary metabolites from living organisms. Some biopesticides, such as those derived from neem (*Azadirachta indica*) are in common use,^4^ but in general, farmer uptake of biopesticides in developing countries remains relatively low. Explanations for this include a lack of technical support and training, lack of investment in research and policy support, and strong marketing by the pesticide industry.^8^ Parsa *et al*.^8^ identified the need for collective action within a farming community as the main obstacle to IPM adoption in developing countries, where farms tend to be small and in close proximity to each other.

Decisions by individual farmers, for example their choice of pest control products, may be influenced by various socio‐economic and demographic characteristics. These include a farmer's level of education and training, and risk perception of pesticide use^9^, ^10^, ^11^; prior experience, including the use of biological controls^10^, ^12^; annual income, land area under cultivation and engagement in extension activities.^12^ Gender is important; one study found that women's decisions were motivated more by health considerations (i.e. maintaining the health of the household and avoiding risks to farm workers). By contrast, men were more influenced by economic (reduced cost of pest control, increased farm productivity/income) and social factors (recognition as an innovative farmer, production of safer commodities for consumers).^10^

This study focuses on commercial biopesticide products that are purchased ‘off the shelf’ and applied to a crop, rather than non‐commercial biological control methods such as classical biological control (‘one‐off’ introduction of an exotic natural enemy), conservation biocontrol (e.g. protecting natural enemy refuges) or homemade concoctions. There is little information on whether any of these registered biopesticides are currently used by smallholders. Dougoud *et al*.^13^ report that even when biopesticides are included in nationally produced pest management decision guides, they are rarely recommended by advisors.

The purpose of this study was to gain insight into farmers' awareness and perception of biopesticides as alternatives to chemical pesticides. The study examined farmers' current pest control practices and risk perception of chemical pesticides, in addition to their willingness to pay (WTP) for alternative products. The research questions explored were: (i) What are farmers' risk perceptions of chemical pesticides and do farmers perceive chemical pesticides as necessary for crop production? (ii) Are farmers aware of any biopesticide products and what are their perceptions of them? (iii) What is the extent of utilisation of biopesticides and what factors influence farmers decision to use such product/s? (iv) If farmers are not using biopesticide products would they be willing to use, and to pay for them, and what factors influence farmers' willingness to pay for such products?

## 2 MATERIALS AND METHODS

### 2.1 Survey site selection and detail

Given that biopesticide use in Kenya was thought to be generally low, sites were selected purposively in areas where it was expected that there might be at least some use and awareness of the products. This was achieved by examining the database of recommendations given at Plantwise (www.plantwise.org) plant clinics in Kenya, of which there are currently more than 200, spread across 20 counties. A search of the database (with permission from Plantwise) identified geographical areas where plant doctors had most frequently made recommendations for biopesticide products; as a result, five counties were selected for this study.

### 2.2 County details

**Figure 1:**
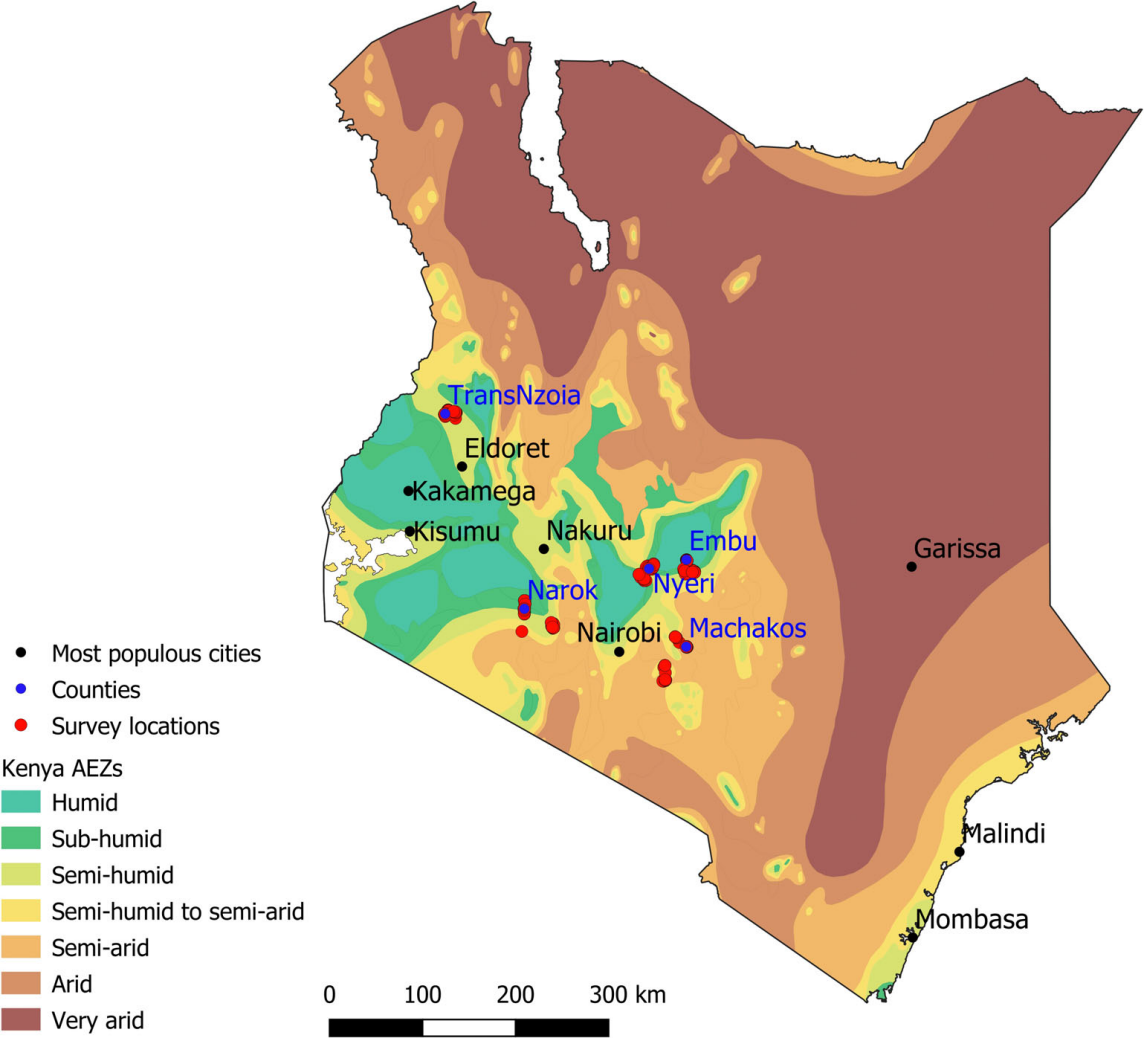
Survey location by agro‐ecological zone

The selected survey areas were within Central (Nyeri), Eastern (Embu, Machakos) and Rift Valley (Narok, Trans Nzoia) Provinces of Kenya. Agro‐ecological zones included humid through to sub/semi‐humid to semi‐arid (Fig. 1). Agriculture is the main economic activity in each of the selected counties (Table 1).

**Table 1:** Agricultural characteristics of each county

County	Agricultural characteristics
Embu	Food crops include maize, beans and other legumes, Irish and sweet potatoes, cassava, arrow root and yams, as well as fruit, vegetables and cereal crops such as millet and sorghum. Cash crops include coffee, tea and Macadamia nuts in the upper part of the county as well as cotton^13^
Nyeri	Renowned for its high production of tea and coffee for export, as well as horticultural farming. Maize, legumes, tubers and vegetables are the main food crops^14^
Machakos	Main economic activity is subsistence farming with maize farming prominent alongside beans, peas, sweet and Irish potatoes, with inclusion of drought‐resistant crops such as sorghum and millet. Cash crops include coffee, cotton and horticultural crops^15^
Narok	Major economic activities include tourism, crop and livestock farming and mining. Main crops are wheat, barley, maize, beans, Irish potatoes and horticultural crops^16^
Trans Nzoia	Located in the Rift Valley, characterised by humid and semi‐humid climate. Main crops include maize, beans, wheat and potato. Tea, coffee, horticulture and commercial businesses are very significant to the economy.^17^

### 2.3 Household surveys

For each county, at least three locations were selected for enumeration, with support from local agricultural extension agents who recommended locations they felt had more exposure to biopesticide use; however, it is acknowledged that the sample may not necessarily be representative of the county. Selection of respondent household per enumeration area followed systematic random sampling, targeting every fifth household as enumerators walked through villages/communities. Face‐to‐face interviews with farmers were conducted by trained enumerators using a structured questionnaire that had been pretested for validity. The questionnaire was programmed on the Open Data Kit (ODK) platform and deployed on tablet computers. At the end of the exercise, 317 smallholders (121 female) had been interviewed (Table 2). The sample size was more than adequate at the 0.05 alpha level and 5% margin of error for categorical data.^18^ Household surveys took place from September to November 2018. The reference season for production data collected was the March to June 2018 cropping season.

**Table 2:** Number of household, extension agent and agro‐dealer interviews and focus group discussions (FGDs) per county

County	No. of household interviews	No. of extension agent interviews	No. of agro‐dealer interviews	No. of FGDs (participants)
Embu	69	5	9	2 (54)
Machakos	67	8	12	2 (25)
Narok	64	5	10	
Nyeri	51	6	11	2 (26)
Trans Nzoia	66	6	10	
Total	317	30	52	6 (105)

### 2.4 Key informant interviews

Interviews using prepared question guides were conducted with 30 extension agents (13 female) and 52 agro‐dealers (20 female) across the survey locations (Table 2). The aim was to determine crop problems presented and control products requested by farmers, recommendations given to farmers, farmer awareness of and recommendations for any biopesticide products, and perceived challenges and factors that would encourage increased smallholder uptake of biopesticides.

### 2.5 Focus group discussions

Two focus group discussions were conducted in Nyeri (Mathira east and Mukurweini), Embu (Gaturi south and Rukira) and Machakos (Kiima kimwe and Kabaa) (Table 2). Focus group participants were purposively selected to include both men and women who were household heads, based in the respective sites, and farmers. To ensure that participants met the criteria, area plant doctors were used to link to appropriate farmers. Facilitators led each focus group through a set of pre‐determined sub‐topics. The topics were centred on: (i) main farming activities and practices; (ii) decision‐making regarding agricultural practices and factors that influence the process; (iii) pest management of key pests and diseases; (iv) biopesticide knowledge, experience and use; and (v) sources of agricultural information. In total, 105 farmers (68 women) participated in the focus group discussions.

### 2.6 Data analysis

For chemical pesticides used by farmers (where the trade name was given), a search was made in the published list of registered products^19^ or online, to determine the active ingredients. Farmers scored perceived risk from the application of chemical pesticides on a five‐point Likert scale: 1, not risky; 2, slightly risky; 3, moderately risky; 4, very risky; and 5, extremely risky. The Risk Index (mean score) and standard deviation (SD) are presented. Farmers scored their level of agreement with four questions about chemical pesticide use on a five‐point Likert scale: −2, strongly disagree; −1, disagree; 0, neutral; 1, agree; and 2, strongly agree. The Acceptance Index (mean score) and SD are presented. For non‐chemical pest control options, chi‐square tests (with Yates's correction) were used to test the significance of differences in proportions between male and female respondents. Farmers' WTP for a biopesticide rather than a chemical pesticide was assessed by asking respondents to choose one of the following options: not willing to pay anything extra; willing to pay 1–5% extra; willing to pay 6–10% extra; willing to pay 11–15% extra; willing to pay 16–20% extra; and willing to pay ≥ 20% extra (current expenditure on chemical pesticides). The proportion of respondents in each category was multiplied by the category's mid‐point to obtain a mean WTP figure with data also disaggregated by gender. A proportional odds model was used to determine what factors influence a farmer's WTP for a potential biopesticide product. WTP was set as the dependent variable and the levels within were ordered in the following condensed categories: not willing to pay; willing to pay 1–10% above current expenditure on a chemical pesticide; and willing to pay ≥ 11% above current expenditure on chemical pesticides. County, gender, age, education, farm size, farm specialisation, proportion marketed, where produce is sold, training in IPM, training in pesticide use, experience of health effects after use of chemical pesticides, and risk to the applicator were used as independent variables within the model. Education, proportion marketed and risk were converted into appropriately ordered factors. An analysis of deviance (type II test) was conducted to determine the overall effect each independent variable had on a farmer's WTP. Tukey's *post hoc* test was used to make a pairwise comparison of categorical variables. The statistical analysis was run in R version 3.5.2,^20^ using packages MASS^21^ and emmeans.^19^

Amounts are indicated in US dollars ($) and were converted from Kenya shillings using a rate of $1 = 102 KES.

## 3 RESULTS

### 3.1 Demographics and farm characteristics

Overall, 62% of respondents were male and 38% female. The majority of interviews were carried out with the household head (74%) or their spouse. The household head (65%) or the household head and spouse jointly (31%) made the farming decisions. Table 3 details farmer characteristics. The average age of respondents was 51 years. There were significant differences between genders in terms of area farmed, level of education, farm specialisation and proportion of produce marketed (*P* < 0.01). Over half of respondents had received training in chemical pesticide use (60%) but fewer had received training in IPM (37%). Almost half of respondents had heard of or used biopesticides (48%) and significantly more men than women had heard of or used biopesticides (*P* < 0.01).

**Table 3:** Farmer characteristics

Characteristics	Overall	Female	Male	*P*‐value
Respondent age (years)	51.2 (0.8)	51.7 (1.3)	50.9 (1.0)	0.631
Household size (no. of members)	5.0 (0.2)	4.8 (0.2)	5.1 (0.2)	0.299
Farmed land (acres)	3.7 (0.4)	1.9 (0.2)	4.8 (0.6)	0.000
Household members working fulltime on farm (no.)	1.9 (0.1)	1.6 (0.1)	2.0 (0.1)	0.084
Distance to nearest agro‐dealer shop (km)	2.8 (0.2)	2.7 (0.2)	2.9 (0.3)	0.621
Distance to nearest market (km)	4.6 (0.4)	4.1 (0.5)	5.0 (0.5)	0.204
Respondent highest level of education (%)				
Primary school	48.6	55.4	44.4	0.000
Secondary school	32.5	35.5	30.6	
Tertiary	15.1	4.1	21.9	
None	3.8	5.0	3.1	
Farm specialisation (%)				
Food crops	41.6	53.7	34.2	0.006
Cash crops	36.0	28.9	40.3	
Livestock	0.6	0.0	1.0	
Mixed enterprise	21.8	17.4	24.5	
Proportion of farm produce marketed				
A minor part (< 20%)	13.6	23.1	7.7	0.000
Moderate (21–60%)	42.0	40.5	42.9	
Most of it (> 60%)	44.5	36.4	49.5	
Where is farm produce marketed (%)				
Local market within the district (subcounty)	54.6	63.6	49.0	0.100
Market within the county	20.8	15.7	24.0	
Farm gate	22.4	19.8	24.0	
Other county	0.3	0.0	0.5	
Market in Nairobi	1.9	0.8	2.6	
Received training on integrated pest management (%)	36.6	40.5	34.2	0.257
Received training on pesticide use (%)	59.9	57.0	61.7	0.406
Received extension service in the last 12 months (%)	68.5	66.9	69.4	0.649
Has access to credit (%)	19.9	19.0	20.4	0.762
Member in farmer organisation (%)	59.0	65.3	55.1	0.073
Heard/used biopesticides (%)	47.9	36.4	55.1	0.001

Figures in parentheses are standard error. Student's t‐ and chi‐squared tests were used to test significance of means and proportions respectively between male and female respondents. Significance is indicated by the *P*‐value.

### 3.2 Chemical pesticide use

Overall, 87% of farmers in the household interviews reported using chemical pesticides to manage various crop pests experienced in the last cropping season. Significantly more males than females used chemical pesticides (*P* < 0.05). Pesticides were used against a range of pests including fall armyworm (*Spodoptera frugiperda* J.E. Smith) (Lepidoptera: Noctuidae), a major invasive pest on maize, thrips (Thysanoptera), cutworms (various species in the Noctuidae) and whitefly (Aleyrodidae), on coffee, Irish potato and kale, respectively. Two of the focus groups reported regular use of chemical pesticides. In Mukurweini, coffee farmers used chemical pesticides and fertilisers as well as crop rotation and cultural methods (e.g. ash, fermented tobacco and smoking). In Kabaa, farmers reported heavy reliance on chemical pesticides while also using other management methods such as crop rotation, sticky traps and pheromone lures.

For their primary crop, 65% of respondents reported using chemical pesticides. The primary crop for 48% of farmers was maize, followed by coffee (8%), Irish potato (7%) and a range of other crops grown by a smaller proportion of farmers. On the primary crop, the most commonly used chemical pesticides contained lambda‐cyhalothrin and alpha‐cypermethrin (Table 4); both were applied primarily to maize (in 13% and 9% of applications), and most frequently against fall armyworm. The WHO^22^ classifies the active ingredients of all but two of these pesticides as moderately hazardous (WHO II classification; see Table 4 for definition of WHO classification levels). An additional 52 chemical products were reported as being used for pest management on the primary crop by a smaller proportion of farmers (≤ 1%) although it was only possible to identify the active ingredients in half of these.

**Table 4:** Active ingredients of chemical pesticides used most frequently on primary crops

Active ingredient	Proportion farmers using (%)	WHO classification^*^
Lambda‐cyhalothrin	19	II
Alpha‐cypermethrin	18	II
Chlorantraniliprole	11	U
Imidacloprid + Beta‐cyfluthrin	5	II + Ib
Chlorpyrifos + Cypermethrin	5	II + II
Carbosulfan	3	II
Diazinon	2	II
Chlorpyrifos	2	II
Diflubenzuron	2	III
Other (used by ≤ 1% of farmers)	32	

* World Health Organisation (WHO, 2010) classification of pesticide active ingredients: Ia, extremely hazardous; Ib, highly hazardous; II, moderately hazardous; III, slightly hazardous; U, unlikely to present acute hazard in normal use.

Farmers who used chemical pesticides reported spraying on average three times in a season for maize, whereas tomato farmers sprayed weekly for the 3 months' cropping cycle. The cost of using chemical pesticides, although not available by individual crop, averaged $43 (± 6.3) per hectare over a cropping season across the five counties. On average farmers in Nyeri spent the most per hectare ($87 ± 23.6) and those in Narok and Trans Nzoia the least ($28 ± 4.1 and 32 ± 5.9, respectively) (Table 5). Disaggregation by gender showed no significant difference in expenditure on pesticides.

**Table 5:** Farmer spending on chemical pesticides

	County					Average
	Nyeri	Embu	Machakos	Narok	Trans Nzoia	
Average spend per cropping season (US$ ha^−1^)	87 (23.6)	76 (8.6)	75 (18.8)	28 (4.1)	32 (5.9)	43 (6.3)

Figures in parentheses are standard errors.

### 3.3 Farmer perceptions of chemical pesticide risk and acceptance

Respondents scored a number of perceived risks of using chemical pesticides on a five‐point Likert scale (Table 6). Farmers perceived the greatest risk of chemical pesticides being to the health of those applying them. There was no significant difference in perception between male and female farmers.

**Table 6:** Farmers' perceived risk of chemical pesticide

Perceived risk	Risk Index
Health of applicators	4.38 (0.7)
Health of other farmers	4.04 (1.0)
Food safety	4.00 (0.9)
Water quality	3.87 (1.0)
Air quality	3.80 (1.0)
Soil quality	3.44 (1.1)
Health of farm animals	3.20 (1.2)
Pest natural enemies	3.17 (1.2)
Plant diversity	2.63 (1.3)
Health of wildlife	2.59 (1.3)

Average farmer rating of risks from application of chemical pesticides (Risk Index) are shown on a five‐point Likert scale: 1, not risky; 2, slightly risky; 3, moderately risky; 4, very risky; and 5, extremely risky. Values are given as mean (SD).

Of farmers who reported using chemical pesticides, 42% stated that someone within their household had experienced negative health effects after applying chemical pesticides in the past 12 months. The most common symptoms reported by those experiencing negative health effects were skin irritation (38%), headaches (28%), dizziness (25%) and stomachache (8%); others reported sneezing, chest problems, fatigue, coughing and sore throat. Farmer awareness and concern of the health aspects of chemical pesticide use were further highlighted by focus group discussions with examples of detrimental health effects that farmers linked to pesticide use including skin rashes, burns, headaches and dizziness. Farmers were asked if they perceived chemical pesticide use necessary for crop production by ranking their responses indicating their level of agreement or disagreement with four questions, the overall mean score providing an Acceptance Index (Table 7).

**Table 7:** Farmer acceptance of chemical pesticide questions

Question	Acceptance Index
1. Do you think pesticides are necessary for crop production?	0.25 (1.7)
2. Do you think the use of pesticides can be harmful to your health?	1.77 (0.6)
3. Do you think that the harmful effects by pesticides can be severe?	1.75 (0.5)
4. Do you think that there could be alternatives to pesticides in crop production?	1.53 (0.8)

Mean farmer responses indicating their level of agreement or disagreement about chemical pesticide use on a five‐point Likert scale: −2, strongly disagree; −1, disagree; 0, neutral; 1, agree; 2, strongly agree. Values are given as mean (SD).

### 3.4 Farmer awareness, perceptions and use of alternative pest control options

A majority of farmers (70%) reported using other pest management methods in combination with chemical pesticides. Almost 60% of farmers used cultural methods such as field sanitation, uprooting and burning infected plant parts, use of resistant varieties, trap cropping, and push–pull methods. Over half (52%) used home‐made plant extracts such as neem, tobacco and hot pepper. Physical/mechanical methods such as hand‐picking were also used, especially for fall armyworm. Some farmers (10%) reported using biopesticide products (including pheromone traps) (Table 8). In some instances, extension agents recommended cultural or physical controls and biopesticides. For example, 18% of recommendations by extension agents for *Tuta absoluta* were for an azadirachtin‐based product, and in half the cases of cutworm damage *B. thuringiensis* was recommended. However, although most extension agents were aware that biopesticide products are available, only 33% reported that they had received training in their use. The two focus groups in Embu reported rarely using chemical pesticides, except for coffee farmers; farmers in Mathira‐east and Kiima kimwe were organic farmers who did not use any chemical pesticides, instead relying on cultural solutions.

**Table 8:** Non‐chemical pest control options used by respondents

Other pest control methods	No. of farmers			χ^2^	*P*‐value
	Overall	Female	Male		
Cultural	187	77	110	1.449	0.229
Plant extracts (farm level)	163	70	93	2.838	0.092
Physical/mechanical, e.g. hand picking	105	36	69	0.773	0.379
Biopesticides	23	5	18	2.136	0.144
Pheromone traps	7	0	7	n/a	n/a
Other	6	3	3	0.032	0.859

Chi‐square (with Yates correction) was used to test significance of proportions between male and female respondents.

**Figure 2:**
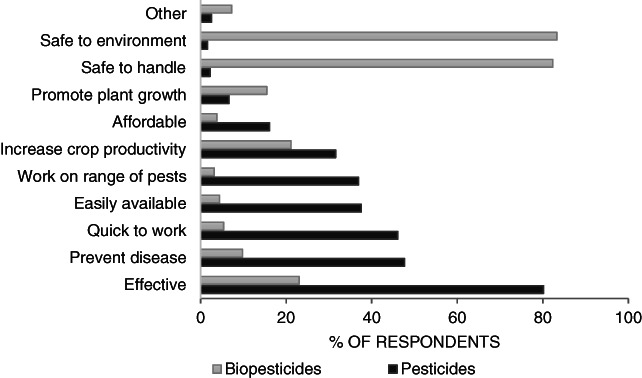
Farmer perceived advantages of chemical pesticides and biopesticides

Almost half (48%) of respondents reported they had heard of or used biopesticides (Table 3). There was limited awareness of specific biopesticide products/active ingredients among interviewed farmers not currently using biopesticides (Table 9). All respondents commented on the perceived advantages of chemical pesticides and biopesticides (Fig. 2). The focus group discussions highlighted low levels of awareness and little or no access to information on biopesticides. Although extension agents felt farmers are aware that biopesticides are effective, farmers also reported perceived limitations, including that products are limited to one pest and are slow acting. Two‐thirds of extension agents stated that farmers perceived biopesticides to be inaccessible to them (financially or physically). Over half (58%) of the agro‐dealers interviewed said they stocked at least one biopesticide product, but subsequent analysis showed that not all of the products referred to were biopesticides. Some were fertilisers and others were unregistered products for which it was not possible to determine the active ingredient. Registered biopesticide products were available at 47% of the agro‐dealer shops in this study (Appendix S1). Agro‐dealers reported low demand and utilisation of biopesticide products by smallholders.

**Table 9:** Awareness of specific biopesticide active ingredients

Biopesticide (product active ingredient)	Percentage of farmers heard of product
Azadirachtin (neem)	29
*Bacillus thuringiensis*	16
*Beauveria bassiana*	13
*Trichoderma* sp. fungus	12
Predatory spider mites	8
Parasitic wasps	6

At the time of this study, 10% of respondents (31 farmers) were using biopesticide products. The most frequently used were products containing azadirachtin, with some use in each of the counties surveyed (Table 10). Three farmers used more than one biopesticide product. In total, 18 respondents using biopesticides were male and 13 were female (58% and 42%, respectively). Farmers' reported that effectiveness, recommendation by extension agents and safety were the main reasons for using biopesticides. The majority of farmers using biopesticides had some level of education, with a high proportion (65%) possessing a higher education (secondary or tertiary). Over two‐thirds (68%) of the farmers using biopesticides were aged 46 years and above. The majority of smallholders (74%) using biopesticides farmed an area of < 2 acres. Sources of agricultural information for those using biopesticides were extension agents (68%), friends and neighbours (65%), radio (52%), agro‐dealers (48%), and farmers' groups (45%).

**Table 10:** Detail on biopesticide use by county

County	Biopesticide active ingredient	Use by gender (*n*)		Pest	Crop	Reason for use	Mean spend per ha/cropping season (US$)
		Male	Female				
Nyeri	Azadirachtin	1		Thrips	Tomato	Effectiveness	48.4
	*B. thuringiensis*	2		Caterpillars	Onion, coffee, tomato	Effectiveness	193.7
	*B. bassiana*	2	1	Aphids	Cabbage	Recommended by extension agent; effective; safe	213.1
	*Trichoderma* sp.	1		Nematodes	Tomato	Available and effectiveness	96.9
Embu	Azadirachtin	1		Not given	Not given	Safety	Not given
	*B. thuringiensis*		1	Caterpillars	Kale	Effective, easy to apply, safe for human and environment	58.1
	*B. bassiana*		1	Whiteflies	Kale	Recommended by extension agent and agro‐dealer	48.4
Machakos	Azadirachtin	2	7	Thrips	Tomato/ capsicum	Recommended by extension agent/agro‐dealer, effective, safety, available, affordable	9.08
	*B. thuringiensis*		1	Caterpillars	Kale	Sold by agrochemical company. Very effective	48.4
Narok	Azadirachtin		1	Aphids	Kale, cabbage	Recommended by extension agent and agro‐dealer	*not given*
	*B. thuringiensis*		1	Stem borer	Maize	Recommended by extension agent	155.0
	*Trichoderma* sp.	4		Blight, soil drenching, PCN	Irish potatoes	Effective, recommended by agro‐dealer/extension agent, affordable, and safe, very effective	65.4
Trans Nzoia	Azadirachtin	5		Fall armyworm, aphids, whiteflies, caterpillars	Maize, kale, cabbage, bean, capsicum	Affordable, available, previously used, recommended by friend/family/agro‐dealer/extension agent, effectiveness, ease of application, safety	48.1
	*B. thuringiensis*	1		Caterpillars	Maize	Recommendation by friend/family, safety	20.6
	*Trichoderma* sp.	3		Wilt disease, soil worms	Tomato, Irish potato	Recommended by friend/family, safety	54.5

PCN, potato cyst nematode.

Data on the cost of biopesticides were provided by some of the farmers (*n* = 18). Calculations were made of the cost per hectare over a cropping season of applying the biopesticides; however, in most instances biopesticide was applied to a specific crop grown over a small area. The average amount spent over a cropping season for the most common biopesticides used was (active ingredients): *B. bassiana*, $131 ha^−1^; *B. thuringiensis*, $95 ha^−1^; *Trichoderma* sp., $72 ha^−1^; and azadirachtin, $35 ha^−1^. Spending on biopesticides varied between counties, although the sample size was small. Overall, the highest biopesticide use intensity ($ ha^−1^) was reported in Nyeri and the least was in Machakos (Table 10).

### 3.5 Willingness to pay for biopesticides

The majority (98%) of farmers currently not using biopesticides stated they would be willing to use these products. The mean amount farmers are willing to pay, above their current expenditure on a chemical pesticide, for a biopesticide product that is just as effective ranged from 8.4 to 10.4% across the five counties, equivalent to $2.7–7.9 ha^−1^ (Table 11). The average WTP across the five counties was 9.6%, which equates to $5.7 above current chemical pesticide expenditures per a cropping season.

**Table 11:** Farmers' willingness to pay (percentage above current expenditure on chemical pesticides) for an effective biopesticide product (cropping season/ha)

County	Female (%)	Male (%)	Mean (%)	US$
Nyeri	3.8	5.3	9.1	7.9
Embu	4.1	6.2	10.4	7.9
Machakos	3.8	5.8	9.6	7.2
Narok	2.7	7.6	10.3	2.9
Trans Nzoia	1.7	6.7	8.4	2.7
Average	3.2	6.3	9.6	5.7

County (location), education level, health perception, and farm specialisation had statistically significant effects on WTP (Table 12). Post‐hoc analysis found that those with a tertiary education were significantly more willing to pay than those with no education (*P*_(0.006)_ < 0.01). In terms of county, respondents from Embu were significantly more willing to pay than those from Trans Nzoia (*P*_(0.008)_ < 0.01). For farm specialisation, more respondents growing a mixed enterprise were willing to pay than those growing food for home consumption (*P*_(0.078)_ < 0.1). Whether farmers had experienced health effects due to chemical pesticides was also a significant predictor of farmers' WTP (*P*_(0.02)_ < 0.05).

**Table 12:** Variables affecting farmers' willingness to pay

Independent variable	LR stat	d.f.	*P*‐value
County	12.884	4	0.012
Gender	0.235	1	0.628
Age	0.903	1	0.342
Education	12.063	3	0.007
Farm size	0.012	1	0.912
Farm specialisation	6.735	3	0.081
Proportion marketed	0.695	2	0.706
Where sold	0.744	4	0.946
Training integrated pest management	0.990	1	0.320
Training pesticides	0.354	1	0.552
Health perception	5.696	1	0.017
Perceived risk to applicator	4.086	4	0.394

## 4 DISCUSSION

The purpose of this study was to contribute to an understanding of why biopesticides are not used more widely, despite increasing focus on IPM and utilisation of low‐risk pest control measures as a component of sustainable agriculture. The study focused on smallholders in Kenya. The assumption on which the study was based, that biopesticides usage is relatively low, was clearly confirmed, with only 10% of farmers using registered products that fall within our broad definition of biopesticides. There are several possible reasons for this, and these are discussed in the light of the study results. Given that a large proportion of farmers use conventional pesticides (87% in the survey), the question can also be framed as why do farmers use conventional pesticides but not biopesticides?

A reason sometimes cited for the low use of biopesticides is that farmers consider biopesticides to be not very effective, or at least less effective than chemical pesticides.^23^ In our survey, 80% of farmers rated conventional pesticides as effective, compared with only 23% for biopesticides. Indeed, when farmers seek chemical pesticides they are looking for quick and effective solutions to protect crops from pests and ensure farm productivity, with their decisions driven by product performance and effectiveness, closely followed by affordability and accessibility (supply by local dealers).^24^ Farmers' perceptions of effectiveness are important, especially considering the risks in trying a new product, from the initial cost of purchase to the subsequent risk of it not being effective against the pest.^25^ With regards to chemical pesticides, farmers' perceptions of efficacy are central to selection and an important predictor of attitudes towards, and knowledge of, IPM and cultural control methods, as well as the importance of pesticide safety.^23^, ^26^ From key informant interviews and focus group discussions, there are some specific elements of biopesticide ‘efficacy’, apart from the overall efficacy at reducing crop loss. One of these is the speed of action. Biopesticides often work more slowly than conventional pesticides, so live pests may still be visible after treatment, even though their feeding rate, and the damage they cause, is much reduced. Over 45% of farmers reported that pesticides are quick to work, but only 5% felt biopesticides work quickly. This is supported by a study in West Africa in which farmers reported measuring effectiveness of pest control by immediate action in terms of insect kill, whereas ‘invisible’ effects that occur after a time delay result in difficulty in evaluation and subsequent reduced likelihood of use.^27^ Thus one approach to promoting the uptake of biopesticides would be to improve farmers’ understanding of the way in which they work. This was also reported in Ghana where a key incentive for choosing a pest control product is cited as speed of action, alongside broad‐spectrum activity and availability.^28^ Not surprisingly, if farmers are used to seeing dead pests following application of a chemical pesticide, the presence of live pests following application of a biopesticide will make it appear as though the treatment has not been effective.

Another aspect of biopesticide efficacy may be that they are more sensitive to correct usage, including factors such as storage, application method and timing. The survey does not give any indication of the extent to which this may result in farmers feeling biopesticides are less effective, and would need a more detailed survey of usage to be conducted.

A further aspect of efficacy which the key informant interviews and focus group discussions indicated reduces the attractiveness of biopesticides is that they can be pest specific. Nearly 40% of respondents reported that chemical pesticides work on a wide range of pests, but fewer than 5% said the same for biopesticides. Again, there may be scope for increasing farmer awareness of biopesticides that are not pest specific. For example, a number of *B. thuringiensis* products are effective against a range of invertebrate pest species.^29^ However, target‐specific products are seen as beneficial by those aiming to reduce environmental damage from crop protection, as non‐target species, including natural enemies of pests, are not affected. But if a farmer is confronted by several pests, buying a product that can manage several or all of them is likely to be preferred over having to buy a separate product for each. Indeed, farmers tend to prefer broad‐spectrum products that are quick to act.^30^ A technical solution to this could be to combine more than one active ingredient in one product, although this might pose additional cost constraints. Some advances have been made in the activity spectra of biopesticides but commercialisation success remains limited.^29^

One reason sometimes cited for the low use of biopesticides is that they are not available, either because they are not registered in a country, or because they are not stocked where farmers buy their pest control products. Indeed, accessibility is cited as an important criteria for chemical pesticide selection and use.^24^ Bateman *et al*.^31^ found that among 19 countries assessed, Kenya had the highest number of biopesticide active ingredients^21^ and registered products (85) that could potentially be used to control fall armyworm. This suggests that the availability of products may not be the major constraint to their use in Kenya. Indeed, registration can be a challenge for biopesticide products because data requirements are often based on those for chemical pesticides, which can be inappropriate.^32^ Nevertheless, fewer than 5% farmers in our survey reported that biopesticides are readily available, which presumably means not all registered products are stocked in retail outlets. In terms of accessibility, interviews with agro‐dealers confirmed that if there is little market for a product, they are less likely to stock it. In this study, 58% of agro‐dealers reported stocking at least one biopesticide, with the most frequently stocked products being those containing azadirachin (stocked by 19% of agro‐dealers). There may be a negative feedback loop in operation; retailers do not stock biopesticides because they perceive there to be low demand, but farmers cannot easily express that demand if biopesticides are not stocked. This is compounded by the fact that biopesticides containing live organisms have a short shelf life, increasing stockist risk if demand is low. Srinvasan *et al*.,^33^ identify availability and access to biopesticides as major constraints to greater adoption in Africa (and Asia). However, there are likely also other reasons for the low demand, so simply getting agro‐dealers to stock more biopesticides might have little impact on sales.

Cost is a consideration when farmers decide what pest control products to purchase.^24^, ^34^ As outlined by Sharifzadeh *et al*.,^24^ when selecting chemical pesticides, financial and accessibility criteria were of high importance for farmers, after performance and effectiveness. Indeed, a number of studies report affordability as a key determinant in the selection and use of conventional pesticides. For example, frequency of application is dependent on the ability to purchase pesticides,^35^ and hesitancy in purchasing costly pesticides is associated with lack of off‐farm income.^24^, ^34^ Particularly in comparison with older generic pesticides, biopesticides may be relatively expensive and this may dissuade farmers from purchasing them. There were too few farmers using biopesticides to get a realistic comparison of costs with conventional pesticides, but other findings from the survey shed some light on this. Only 4% of farmers reported biopesticides as being affordable, although only 16% of farmers said chemical pesticides are affordable, even though a high proportion of farmers purchase them. Thus, farmers may be expressing something to the effect that ‘pesticides are expensive but still worth it, but biopesticides aren't’, linking back to the perceptions of efficacy. This is confirmed by the farmers' WTP for a biopesticide product of equal efficacy to a conventional pesticide. Farmers expressed an average WTP for biopesticides of 9.6% above their current expenditure on chemical pesticides for an equally effective biopesticide product. The average WTP figure is low compared with similar studies,^36^, ^45^ but not to others, which is not surprising. In a similar study in Pakistan, farmers were willing to pay to avoid pesticide health risks, but the mean WTP was 8.1% of pesticide expenditures.^36^ Adetonah *et al*.,^37^ also found low WTP, with the amount farmers are willing to pay highly dependent on income and resource availability. However, even low WTP reflects the fact that resource‐poor farmers are willing to consider effective alternative pest solutions. This is supported by Coulibaly *et al*.^28^ who report ‘WTP for a new biopesticide is driven principally by the perception that current synthetic insecticides are ineffective, and that there is a need for alternatives’. Reduced cost and/or subsidies for biopesticide products could be a strategy to facilitate greater uptake while allowing biopesticide companies/products to secure a foothold in the market and enhance their ability to compete with established chemical pesticide products.^38^ The high dependence of farmers on chemical pesticides for fall armyworm management in Zambia was attributed, in part, to government provision of free supplies.^39^

One approach sometimes used to promote biopesticides to farmers is that they are safer than using chemical pesticides, the implicit assumption being that farmers are unaware of the risks and that once they are aware, they will buy biopesticides instead. The results of this survey do not support that assumption. A large proportion of the interviewed farmers (>80%) felt biopesticides are safe to handle and safe for the environment, but fewer than 5% felt similarly about pesticides. Corroborating this, almost all farmers agreed or strongly agreed that chemical pesticides can be harmful to human health. Farmers therefore continue using chemical pesticides despite an awareness of the risks, although farmers who had experienced health problems attributed to pesticides were more willing to pay for biopesticides than those who had not. Other studies report that farmers who have experienced adverse health effects due to chemical pesticide use demonstrate greater concern over health, higher risk perception of unsafe use of pesticides and greater willingness to use biopesticides.^25^, ^40^, ^41^

A further reason why farmers buy conventional pesticides rather than biopesticides may be that they are advised to do so. Pesticide dealers are an important source of advice for farmers. In this study, agro‐input dealers stated all clients request advice on what products to use. The importance of agro‐dealers in the provision of information to farmers has been highlighted previously.^7^, ^27^, ^28^ Pesticide dealers are more likely to recommend products that they stock and which they know farmers consider to be efficacious. Even extensionists are more likely to recommend products that they know are easily available and likely to be effective. Dougoud *et al*.^42^ found that even trained extensionists (plant doctors) do not regularly recommend biopesticides, and this was confirmed by key informant interviews and focus group discussions in this survey. Education, training and extension services, as well as government policy promoting IPM practices, have proved important in increasing biopesticide uptake by vegetable producers.^28^

Although there are several potential entry points through which use of biopesticides can be promoted, from this survey the most important issue is farmer perception of the effectiveness of biopesticides. Farmers’ perceptions may be inaccurate, in that even highly efficacious biopesticides may appear to be less effective than conventional pesticides due to their speed of action. Tackling this requires significant communication and awareness activities among farmers, as well as those providing advice. Of course there may also be situations in which a biopesticide is simply less effective than its chemical counterpart and this is where decision‐making on what products should be used becomes important. It is a challenge to researchers to come up with highly effective but lower risk products. While some of these may already be registered, they may not be readily available and/or may be priced higher than pesticides. Making these products readily available at a competitive price, combined with awareness raising, would be another approach to promote uptake. Although biopesticides currently cover only ~ 4 % of the global pesticide market, they could play a significant role in IPM strategies, particularly considering biopesticide compatibility with a range of other pest management approaches.^43^, ^44^

## 5 CONCLUSION

This study confirms the low use of biopesticide products in the survey areas alongside high use of conventional chemical pesticides. Despite Kenya having a reasonably high number of registered biopesticide products, demand and local availability for these was low, likely resulting in a negative feedback loop. Farmers reported biopesticides to have low efficacy, perceiving them to be slow to work and limited to one pest. Cost is also an issue; farmers perceived both chemical pesticides and biopesticides as expensive, but they still purchase the former despite high awareness of the health implications. Although there was WTP for biopesticide products, this was relatively low. The key to increasing smallholder uptake of biopesticides is to address farmers' perceptions of effectiveness. Farmers rely heavily on advice about what products to use, therefore increasing the knowledge of those providing advice is essential, in addition to ensuring products that are already registered are locally available at competitive prices. These results are reflective of a typical smallholder in the survey counties, and highlight the need for greater awareness and understanding of farmer perceptions because they are central to facilitating the wider use of biopesticides.

## Supporting information


**Appendix S1.** Supporting information
