# Supplementary material for: Why don't smallholder farmers in Kenya use more biopesticides?
Source: Pest Manag Sci. 2020 May 30;76(11):3615–25. doi: 10.1002/ps.5896 (PMC7586800; doi:10.1002/ps.5896)
Supplement: Supplementary file 1 — Appendix S1. Supporting information [file PS-76-3615-s001.docx]

**Annex 1: Registered active ingredients of biopesticide products in Kenya**

| **#** | **Registered active ingredient** | **No. products available** | **No. distributors** | **% agro-dealers stocking in study** | **#** | **Registered active ingredient** | **No. products available** | **No. distributors** | **% agro-dealers stocking in study** |
| --- | --- | --- | --- | --- | --- | --- | --- | --- | --- |
| 1 | *Amblydromalus limonicus* | 1 | 1 |  | 31 | *Helicoverpa armigera SNPV* | 1 | 1 |  |
| 2 | *Amblyseius andersoni* | 1 | 1 |  | 32 | *Hydrolysed Protein 55g/L* | 1 | 1 |  |
| 3 | *Amblyseius californicus* | 1 | 1 |  | 33 | *Hypoaspis miles* | 1 | 1 |  |
| 4 | *Amblyseius cucumeris* | 3 | 3 |  | 34 | *Lecanicillium lecanii* | 2 | 2 |  |
| 5 | *Amblyseius swirskii* | 1 | 1 |  | 35 | *Lecanicillium muscarium* | 1 | 1 |  |
| 6 | *Ampelomyces quisqualis* | 1 | 1 |  | 36 | *Matrine* | 2 | 2 |  |
| 7 | *Aphidius transcaspinus* | 1 | 1 |  | 37 | *Metarhizium anisopliae* | 3 | 2 |  |
| 8 | *Azadirachtin* | 7 | 6 | 21 | 38 | *Methyl Eugenol* | 1 | 1 | 4 |
| 9 | *Azadirachtin 0.6% + Matrine 0.4%* | 1 | 1 |  | 39 | *Myrothecium verrucaria* | 1 | 1 |  |
| 10 | *Bacillus subtilis BS-01* | 1 | 1 |  | 40 | *Naringin* | 1 | 1 |  |
| 11 | *Bacillus thuringiensis* | 4 | 4 |  | 41 | *Neoseilus californicus (Amblyseius californicus)* | 1 | 1 |  |
| 12 | *Bacillus thuringiensis var. kurstaki* | 3 | 2 | 2 | 42 | *Orange oil (Limonene)* | 1 | 1 |  |
| 13 | *Bacillus thuringiensis var. kurstaki Strain ABTS-351* | 1 | 1 |  | 43 | *Oxymatrine* | 2 | 2 | 2 |
| 14 | *Bacillus thuringiensis var. kurstaki, Strain SA-11* | 1 | 1 |  | 44 | *Paecilomyces fumosoroseus strain FE 9901* | 1 | 1 |  |
| 15 | *Beauveria bassiana* | 2 | 2 |  | 45 | *Paecilomyces lilacinus* | 3 | 3 | 2 |
| 16 | *Beauveria bassiana strain GHA* | 2 | 2 |  | 46 | *Paraffinic oil* | 3 | 3 |  |
| 17 | *Benzoic acid* | 1 | 1 |  | 47 | *Petroleum oil* | 2 | 1 |  |
| 18 | *Biological Cotton seed oil 31% + Garlic extract 23%* | 1 | 1 | 2 | 48 | *Phytoseiulus persimilis* | 3 | 3 |  |
| 19 | *Chrysophanol* | 1 | 1 |  | 49 | *Pseudomonas fluorescens* | 1 | 1 |  |
| 20 | *Citric acid* | 1 | 1 |  | 50 | *Pyrethrin* | 7 | 2 |  |
| 21 | *Coccidoxenoides perminutus* | 1 | 1 |  | 51 | *Pyrethrin + Azadiracthin* | 1 | 1 |  |
| 22 | *Cryptolaemus montrouzieri* | 1 | 1 |  | 52 | *Pyrethrin + Garlic extract* | 1 | 1 | 2 |
| 23 | *Diglyhus isaea* | 1 | 1 |  | 53 | *Steinernema carpocapsae* | 1 | 1 |  |
| 24 | *E3, Z8, Z11-tetradecatrienyl acetate 0.5mg + E3, Z8-tetradecadienyl acetate 0.2mg* | 1 | 1 |  | 54 | *Steinernema feltiae* | 2 | 2 |  |
| 25 | *Encarsia formosa* | 1 | 1 |  | 55 | *Thyme oil* | 1 | 1 |  |
| 26 | *Eretmocerus eremicus* | 1 | 1 |  | 56 | *Trichoderma asperellum* | 2 | 2 |  |
| 27 | *Eugenol* | 1 | 1 |  | 57 | *Trichoderma harzianum* | 1 | 1 |  |
| 28 | *Garlic extract* | 1 | 1 |  | 58 | *Trichoderma harzianum Rifai strain KRL-AG2* | 1 | 1 | 12 |
| 29 | *Geraniol, Thymol, Eugenol* | 1 | 1 |  | 59 | *Trichoderma harzianum strain 21* | 1 | 1 |  |
| 30 | *Gibberelic acid* | 3 | 3 |  | 60 | *Trichoderma viride* | 1 | 1 |  |
|  | *Data obtained from PCPB website (PCPB, 2018)* |  |  |  |  | **Total:** | **97** | **29** | **47%** |
